# A review of the UK and British Channel Islands practical tidal stream energy resource

**DOI:** 10.1098/rspa.2021.0469

**Published:** 2021-11

**Authors:** Daniel Coles, Athanasios Angeloudis, Deborah Greaves, Gordon Hastie, Matthew Lewis, Lucas Mackie, James McNaughton, Jon Miles, Simon Neill, Matthew Piggott, Denise Risch, Beth Scott, Carol Sparling, Tim Stallard, Philipp Thies, Stuart Walker, David White, Richard Willden, Benjamin Williamson

**Affiliations:** ^1^ School of Engineering, Computing and Mathematics, University of Plymouth, Plymouth PL4 8AA, UK; ^2^ School of Engineering, Institute for Infrastructure and the Environment, The University of Edinburgh, Edinburgh EH8 9YL, UK; ^3^ Sea Mammal Research Unit, Scottish Oceans Institute, University of St Andrews, St Andrews KY16 8LB, UK; ^4^ School of Ocean Sciences, Bangor University, Menai Bridge LL59 5AB, UK; ^5^ Department of Earth Science and Engineering, Imperial College London, London SW7 2AZ, UK; ^6^ Department of Engineering Science, University of Oxford, Oxford OX1 3PJ, UK; ^7^ The Scottish Association for Marine Science, Oban PA37 1QA, UK; ^8^ School of Biological Sciences, University of Aberdeen, Aberdeen AB24 2TZ, UK; ^9^ Department of Mechanical, Civil and Aerospace Engineering, University of Manchester, Manchester M1 3BB, UK; ^10^ Renewable Energy Group, CEMPS, University of Exeter, Penryn Campus, Penryn TR10 9FE, UK; ^11^ School of Engineering, University of Southampton, Southampton SO17 1BJ, UK; ^12^ Environmental Research Institute, North Highland College, University of the Highlands and Islands, Thurso KW14 7EE, UK

**Keywords:** tidal stream power, tidal stream energy, practical resource, cost of energy, system integration, environmental impact

## Abstract

This review provides a critical, multi-faceted assessment of the practical contribution tidal stream energy can make to the UK and British Channel Islands future energy mix. Evidence is presented that broadly supports the latest national-scale practical resource estimate, of 34 TWh/year, equivalent to 11% of the UK’s current annual electricity demand. The size of the practical resource depends in part on the economic competitiveness of projects. In the UK, 124 MW of prospective tidal stream capacity is currently eligible to bid for subsidy support (MeyGen 1C, 80 MW; PTEC, 30 MW; and Morlais, 14 MW). It is estimated that the installation of this 124 MW would serve to drive down the levelized cost of energy (LCoE), through learning, from its current level of around 240 £/MWh to below 150 £/MWh, based on a mid-range technology learning rate of 17%. Doing so would make tidal stream cost competitive with technologies such as combined cycle gas turbines, biomass and anaerobic digestion. Installing this 124 MW by 2031 would put tidal stream on a trajectory to install the estimated 11.5 GW needed to generate 34 TWh/year by 2050. The cyclic, predictable nature of tidal stream power shows potential to provide additional, whole-system cost benefits. These include reductions in balancing expenditure that are not considered in conventional LCoE estimates. The practical resource is also dependent on environmental constraints. To date, no collisions between animals and turbines have been detected, and only small changes in habitat have been measured. The impacts of large arrays on stratification and predator–prey interaction are projected to be an order of magnitude less than those from climate change, highlighting opportunities for risk retirement. Ongoing field measurements will be important as arrays scale up, given the uncertainty in some environmental and ecological impact models. Based on the findings presented in this review, we recommend that an updated national-scale practical resource study is undertaken that implements high-fidelity, site-specific modelling, with improved model validation from the wide range of field measurements that are now available from the major sites. Quantifying the sensitivity of the practical resource to constraints will be important to establish opportunities for constraint retirement. Quantification of whole-system benefits is necessary to fully understand the value of tidal stream in the energy system.

## 1. Introduction

The UK generates approximately 308 TWh of electricity a year [1]. Of this, 40% is produced using fossil fuels, such as coal and natural gas, which contribute more than 20% of the UK’s annual greenhouse gas emissions [2]. In 2019, the UK legislated net-zero greenhouse gas emissions by 2050, necessitating the replacement of carbon-emitting electricity generation technologies with clean alternatives. The Climate Change Committee’s sixth carbon budget estimates that electricity demand will increase to between 550 and 680 TWh/year by 2050, driven predominantly by the electrification of transport and heating [3]. Wind and solar photovoltaic (PV) generation are estimated to contribute 430 TWh and 85 TWh/year, respectively, totalling 515 TWh/year. Complementary technologies are required to make up the remaining shortfall in supply, and overcome the grid management challenges that increasing levels of variable power production will present, such as balancing.

**Figure 1.**
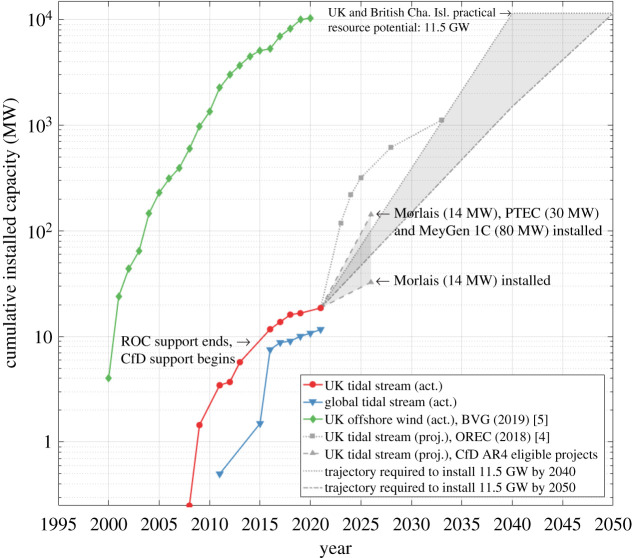
Actual and projected cumulative installed capacity of tidal stream and fixed-bed offshore wind in the UK and globally (excluding the UK).

Tidal stream turbines harness the power of the tides, typically using horizontal axis rotors to drive a generator. Since 2008, 18 MW of tidal stream capacity has been installed in the UK. Of this, 10.4 MW is operational, with the remaining 7.7 MW now decommissioned, having completed testing [4]. The growth in UK tidal stream cumulative installed capacity is shown in figure 1, alongside progress globally, and that of UK fixed-bed offshore wind [5]. The emergence of operational tidal stream projects has been dependent on access to government subsidy. Between 2008 and 2015, tidal stream was supported by the Renewable Obligations Certificate (ROC) scheme. Electricity suppliers purchase ROCs from renewable power generators to fulfil their obligation to provide renewable electricity, while providing the generator with an income per unit of energy supplied [6]. The contracts for difference (CfD) scheme was introduced in 2015 to replace ROCs. The CfD scheme protects generators from volatile wholesale electricity prices by providing a flat rate for electricity production to a renewable power generator, known as the strike price, over 15 years. Developers can apply for CfD support through biennial auction rounds (ARs), where projects with the lowest strike price are selected for CfD support. The first three CfD rounds since 2015 (AR1–3) have provided subsidy support for approximately 11.2 GW of installed capacity; 10.8 GW has been won by fixed-bed offshore wind projects [7–9], which have a significantly lower strike price, enabled through earlier adoption along with steady subsidy support. To date, tidal stream projects, which currently have a relatively high strike price, have not been able to secure CfD support. This has slowed the rate of tidal stream deployment since 2015 significantly, as shown figure 1.

To be eligible to bid in CfD ARs, projects must have secured a lease plot, grid connection and consents. For AR4, scheduled for late 2021, three tidal stream projects are eligible to bid: Morlais in Wales (14 MW), the Perpetuus Tidal Energy Centre (PTEC) in England (30 MW) and MeyGen 1C in Scotland (80 MW). Projects that win subsidy support in AR4 must be operational from 2026. The projected UK cumulative installed capacity, as a result of these projects being built out, is shown in figure 1, alongside a projection by the Offshore Renewable Energy Catapult (OREC) that is discussed in §3. The UK and British Channel Islands estimated tidal stream installed capacity potential is 11.5 GW, which is discussed further in §2. Figure 1 shows that, for 11.5 GW to be installed by 2050, the deployment trajectory must return to its pre-2015 level to reach a cumulative installed capacity of approximately 60 MW (i.e. a combination of Morlais, PTEC and/or MeyGen 1C) before 2027 and 140 MW (i.e. Morlais, PTEC and MeyGen 1C) before 2031. Details of tidal stream installations, both in the UK and elsewhere, are provided in table 1. In general, information on the energy/power performance of operational projects is scarce. Based on the performance data that are available, UK projects have demonstrated relatively low levels of inclusive capacity factor (<0.2), which we define here as the ratio of the energy yield achieved since the beginning of operation to the energy yield that would be achieved if the turbine(s) operate at rated power continuously over the same time period. The inclusive capacity factor does not neglect periods of turbine downtime, as is often done in conventional capacity factor estimation. Low inclusive capacity factor performance is partly down to the fact that, to date, most operational turbines have been deployed for testing purposes, with lower performance targets than commercial projects. Over the development of some 58 tidal stream projects globally since 2003, there has been a marked improvement in turbine reliability, achieved through learning from early-stage turbine deployments at lower flow sites [10]. Verdant Power has reported energy yield figures of 0.3 GWh over a nine-month period, equivalent to a capacity factor of 0.42 [11]. Demonstrating the commercial viability of tidal stream relies on further evidence of sustained high-power performance.

**Table 1.** Tidal stream installed capacity in the UK and globally.

developer	project/site	turbine model(s)	rotors	start of operation	installed capacity	active	energy yield	inc. capacity factor
UK projects								
Orbital Marine Power*	EMEC testing	O2	2	2021	2.00 MW	yes	n.a.	n.a.
Minesto	Holyhead Deep Phase 1	DG500	1	2019	0.50 MW	yes	n.a.	n.a.
Magallanes	EMEC testing	ATIR	2	2018	2.00 MW	yes	n.a.	n.a.
Nova Innovation	Shetland Tidal Array	M100-D	4	2018	0.40 MW	yes	n.a.	n.a.
Orbital Marine Power*	EMEC testing	SR2000	2	2017	2.00 MW	no	3.3 GWh	0.10${\;}^{†}$
MeyGen	MeyGen 1A	HS1500, AR1500	4	2016	6.00 MW	yes	37.0 GWh	0.16${\;}^{‡}$
Tidal Energy Ltd	Ramsey Sound	Deltastream	1	2015	0.40 MW	no	n.a.	n.a.
Alstom	EMEC testing	Deepgen	1	2013	1.00 MW	no	1.2 GWh	0.07${\;}^{⊥}$
Voith Hydro	EMEC testing	HyTide 1000	1	2013	1.00 MW	no	n.a.	n.a.
Orbital Marine Power*	EMEC testing	SR250	2	2012	0.25 MW	no	n.a.	n.a.
SIMEC Atlantis Energy	EMEC testing	AR1000	1	2011	1.00 MW	no	n.a.	n.a.
Andritz Hydro Hammerfest	EMEC testing	HS1000	1	2011	1.00 MW	no	n.a.	n.a.
Marine Current Turbines (MCT)	Strangford Lough testing	SeaGen	2	2009	1.20 MW	no	11.6 GWh	0.10${\;}^{⊺}$
global projects								
OpenHydro	EMEC testing	n.a.	1	2008	0.25 MW	no	n.a.	n.a.
SIMEC Atlantis Energy	Naru Strait, Japan	AR1500	1	2021	0.50 MW	yes	n.a.	n.a.
Sustainable Marine Energy/Schottel	Pempa’q Instream Tidal Energy project, Grand Passage, Canada	PLAT-I 6.40	6	2020	0.42 MW	yes	n.a.	n.a.
Verdant Power	The RITE Project, East River, New York, USA	Gen5	3	2020	0.11 MW	yes	0.3 GWh	0.42${\;}^{≀}$
SIMEC Atlantis Energy	Zhoushan archipelago, China	SG500	1	2020	0.50 MW	yes	n.a.	n.a.
Minesto	Vestmannasund, Faroe Islands	DG100	2	2020	0.10 MW	yes	n.a.	n.a.
HydroQuest	Paimpol-Brehat, France	n.a.	4	2019	1.00 MW	yes	n.a.	n.a.
Sustainable Marine Energy	Digby Neck, Canada	PLAT-I 4.63	4	2018	0.28 MW	yes	n.a.	n.a.
Tocardo	Eastern Scheldt, Netherlands	T-2	5	2017	1.25 MW	yes	n.a.	n.a.
OpenHydro	Paimpol-Brehat, France	L’Arcouest	2	2016	4.00 MW	no	n.a.	n.a.
OpenHydro	FORCE, Canada	n.a.	1	2016	2.00 MW	no	n.a.	n.a.
Sabella	Fromveur Passage, France	D10	1	2015	1.00 MW	yes	n.a.	n.a.
OpenHydro	Paimpol-Brehat, France	n.a.	1	2011	0.50 MW	no	n.a.	n.a.

Inclusive capacity factors estimated based on the energy yield achieved between the following dates: ${\;}^{†}$SR2000, October 2016–September 2018; ${\;}^{‡}$MeyGen 1A, December 2016–July 2021; ${\;}^{⊥}$Alstom DeepGen, January 2013–December 2014; ${\;}^{⊺}$MCT SeaGen, July 2008–July 2019; ${\;}^{≀}$Verdent Gen5, 9-month period (dates not available). EMEC, European Marine Energy Centre; n.a., not available. *Formerly ScotRenewables.

In 2020, the UK government's Department for Business, Energy and Industrial Strategy (BEIS) and the UK Parliament Environmental Audit Committee issued calls for evidence to assess the contribution that tidal stream can make to the UK’s future power generation mix [12,13]. Based on the evidence submitted, the Environmental Audit Committee concluded that ‘there is substantial potential for the tidal sector to make a significant and distinct contribution to the UK’s future mix of energy generated from renewable sources’ [14]. Since the call, BEIS has announced that, for the first time, AR4 will allow tidal stream to compete for subsidy support against other less established technologies, such as floating offshore wind, without competition from fixed-bed offshore wind [15]. The implications of this change are explored in §3b.

**Figure 2.**
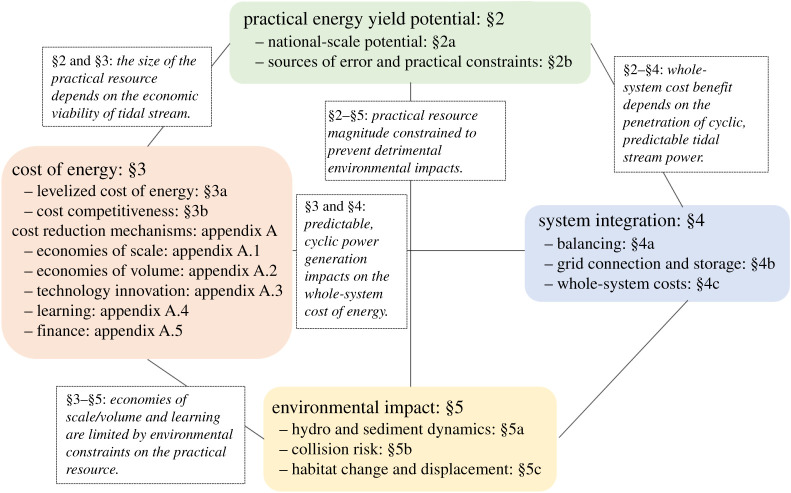
Summary of review topics, with examples of how they are related to one another.

This review expands on information submitted to the BEIS and Environmental Audit Committee calls for evidence by the authors. We focus on the current state of the art in UK practical tidal stream energy resource quantification. The practical resource is defined as the annual energy yield potential that can be harnessed using tidal stream turbines, once consideration for economic, environmental, regulatory and social constraints have been imposed [16,17]. The validity of the latest national-scale practical resource estimates are reviewed in §2. This includes discussion regarding constraints that limit the practical resource, such as those arising from regulation (e.g. laws/regulations that enable/prohibit the use of marine areas), and social activity, such as navigation. The economic viability of tidal stream is reviewed in §3, with consideration for levelized cost of energy (LCoE) and the cost competitiveness of tidal stream relative to other technologies. A summary of cost reduction drivers is also provided in appendix A. The cost competitiveness and therefore practical resource are also dependent on the ease with which tidal stream projects can integrate with the grid and complement other technologies, such as energy storage. These aspects are explored in §4. The environmental impacts of large-scale array development are critical in assessing the practical viability of future tidal stream projects. Impacts such as changes to sediment dynamics, collision risk with marine animals and habitat change are reviewed in §5. Examples of the linkages between these practical resource considerations are summarized in figure 2, and discussed throughout the review.

## 2. Practical energy yield potential

### (a) National-scale potential

**Figure 3.**
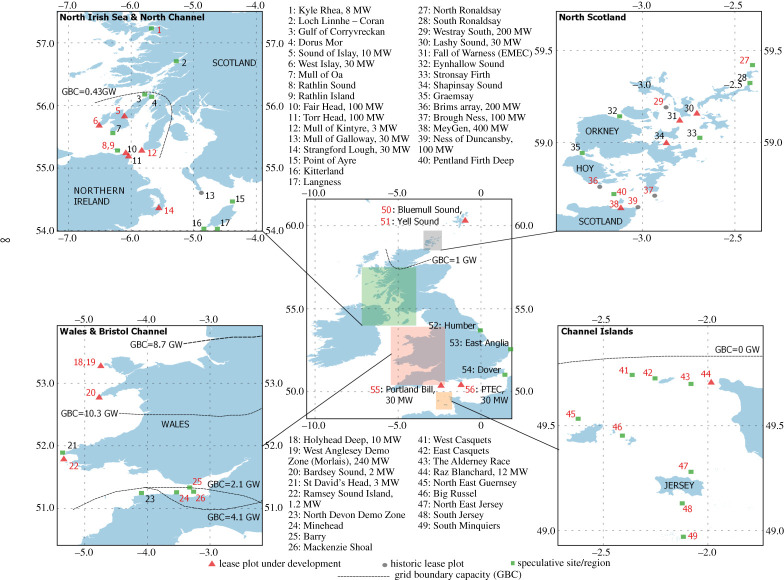
Overview of prospective tidal stream energy lease sites and speculative sites around the UK and British Channel Islands. Red triangles indicate lease plots currently under development, grey circles indicate historic plots that have been withdrawn and green squares indicate speculative sites/regions. Red numbers indicate sites/regions considered in the 2011 Carbon Trust study [16]. Relevant electrical grid boundary capacities (GBCs) are also illustrated.

The Carbon Trust commissioned the latest UK-wide tidal stream practical resource study in 2011 [16]. The study used the shallow-water two-dimensional (2D) hydrodynamic model Tidal Flow Development-2d (TFD-2D) [18] to simulate generic hydraulic current, resonant basin and tidal streaming sites. The model domains (e.g. channel length/width/depth) were modified to approximately match the geometry of 31 sites. The locations of the sites are shown in figure 3, alongside other sites and lease plots that are discussed in this review. The models were forced by the principal semi-diurnal lunar and solar constituents, M2 and S2, respectively. Sites were selected for the study if they exhibited depths greater than 15 m and an estimated mean annual power density that exceeded $1.5\;{\text{kW m}}^{-2}$. These criteria were set based on the conditions required for the economic viability of operational tidal stream turbines at the time. An additional drag term was implemented in the momentum equations to simulate the impacts of blockage caused by tidal stream turbine rotors on the surrounding flow field. Additional drag sources from infrastructure such as the support structures were excluded from the analysis. Constraints on changes to the flow regime (i.e. tidal range and flow speeds) and grid and array spatial extent were implemented to establish practical limits on energy extraction.

The initial estimated practical resource potential, of 21 TWh/year, is equivalent to 6.5% of the UK’s annual electricity demand and a time-averaged annual power output of 2.4 GW. The Carbon Trust's study re-estimated the practical resource based on relaxed cost constraints applied to the tidal streaming and Pentland Firth sites. It was argued that high levels of development in the Pentland Firth region would enable favourable economic mechanisms, such as greater economies of volume, relative to smaller sites (see appendix A for a discussion on cost-reduction mechanisms). Environmental constraints were also relaxed on all tidal streaming sites, on the grounds that the generic hydrodynamic models used were not representative of the ‘open-sea’ sites considered in the study. The re-estimated practical resource, of 34 TWh/year, is equivalent to 11% of the UK’s current annual electricity demand.

The significant increase in the practical resource from this re-estimate (i.e. from 21 TWh/year to 34 TWh/year) highlights high sensitivity to the economic and environmental constraint limits. The Carbon Trust's study acknowledges that sensitivity testing of the arbitrarily prescribed practical limits on energy extraction is required on a site-by-site basis, including improved understanding of the acceptable ambient flow changes, given that they have no regulatory basis. The validity of assumptions regarding constraint setting are now discussed.

**Figure 4.**
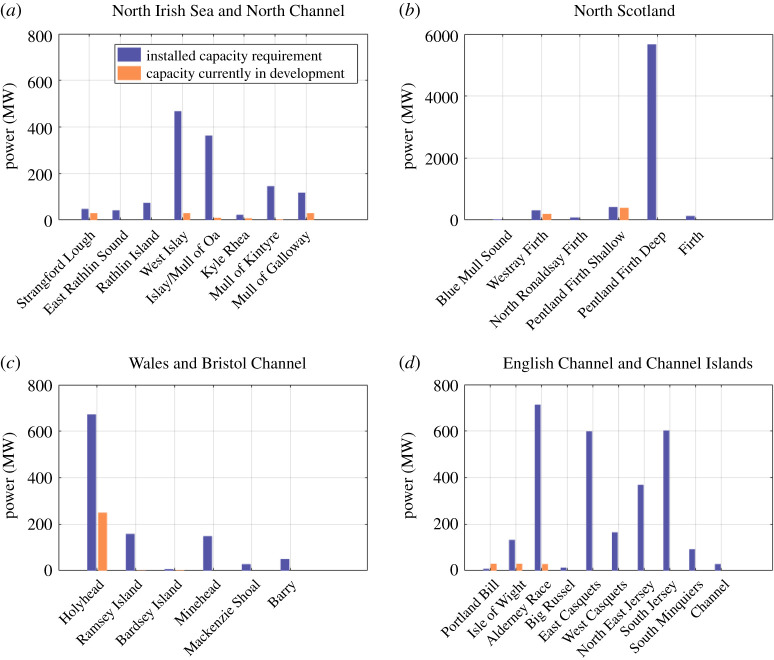
Installed capacity requirement to achieve a practical resource of 34 TWh/year with a capacity factor of 0.34, based on sites used in the 2011 Carbon Trust's study [16]. Note that (*b*) North Scotland has a different *y*-axis limit, given its larger resource.

Figure 4 quantifies the estimated installed capacity required to achieve the Carbon Trust's 34 TWh/year yield. These are compared with the capacity currently under development at each site. In the case of the Alderney Race, the installed capacity requirement has been halved, since half of the Race is located in French territorial waters. Site locations are shown in figure 3, along with others identified around the UK and British Channel Islands. The required installed capacities were estimated based on an inclusive capacity factor of 0.34, which considers all downtime and system losses between the turbines and the grid connection [19].

In the Pentland Firth, 6 GW of installed capacity is required. Subsequent hydrodynamic modelling of the Pentland Firth simulated a 4.2 GW array, demonstrating that a capacity factor of 0.39 (i.e. without downtime and system losses) may be achievable [20]. This work also demonstrated that some environmental impacts caused by the array, such as increased stratification, are an order of magnitude lower than those caused by global warming. Environmental impacts are explored further in §5. The total area of the Pentland Firth site used in the Carbon Trust's study is $260\;{\text{km}}^{2}$. A 6 GW array covering 260 km^2^ has an array density of $23\;{\text{MW km}}^{-2}$. This is equivalent to a lateral and longitudinal spacing between turbines of 8 rotor diameters and 25 rotor diameters, respectively, based on the specification of the MeyGen 1A turbines. This is significantly higher than the minimum lateral and longitudinal spacing recommended by the European Marine Energy Centre (EMEC), of 2.5 rotor diameters and 10 rotor diameters, respectively [21]. These findings support the Carbon Trust's approach to relax economic and environmental constraints imposed on the Pentland Firth region; however, further investigation is required to quantify (i) the potential impacts of constraints that were neglected in the Carbon Trust's study on the practical resource and (ii) the magnitude of errors in the practical resource estimate. In §2b, we quantify the latter.

### (b) Sources of error and practical constraints

The Carbon Trust's practical resource estimates have a reported uncertainty of −50%/+20%. Since the time of the study, research developments have brought to light the potential contribution of errors arising from the methods adopted by the Carbon Trust. These are as follows:

— The hydrodynamic models use M2 and S2 forcings only. This accounts for around 93% of the tidal stream energy [22,23], resulting in an energy yield under-estimation of approximately 7%.
— Energy yield was estimated based upon ‘mean spring flow’ and ‘mean neap flow’ speeds. This method has since been shown to under-predict the energy resource by up to 25% [23].
— The study excluded sites owing to unavailability of field data, including the Point of Ayre, Langness and Kitterland in the Isle of Man, which have been considered for development with a combined installed capacity of 210 MW. Additionally, Stronsay Firth (measured peak flow speeds of $2\;{\text{m s}}^{-1}$ and depths of 30–35 m [24]), Dorus Mor (high-energy site with maximum flow speeds of $4.1\;{\text{m s}}^{-1}$), Orkney Papa Westray, Eday Sound (understood to be Lashy Sound, a 30 MW site currently under development) and Yell Sound in Shetland [25] were not included. An additional 21 relatively low-capacity sites have been identified as potentially suitable for tidal stream energy development, and are shown in figure 3. Based on the proposed install capacities of the Isle of Man sites, they are expected to increase the practical resource by at least 2.5%.
— The accuracy of the approach to represent real sites by generic tidal streaming, hydraulic current and resonant basin domains was investigated, using Strangford Lough as a test case [26]. It was shown that the generic domain simulation under-estimated the resource by 10%, compared with a hydrodynamic model that used the specific site geometry, bathymetry and forcings. Only partial validation of the TFD-2D models was undertaken, as, at the time of the study, field data availability was limited.
— The estimates do not consider the support structure drag of the turbines, which, if included, would increase the blockage effects of the arrays, resulting in greater levels of flow diversion away from the turbines and hence a reduction in energy yield [27,28]. Haverson *et al.* [29] parameterized the support structure drag of monopiles in a hydrodynamic model that simulated an array of turbines located at St David’s Head in Wales [29]. Adopting the same rotor and drag parameterization, with a monopile drag coefficient of 0.9, monopile diameter of 2 m and exposed monopile area of $15\;{\text{m}}^{2}$, we estimate that the contribution of support structure drag, as a percentage of the total device drag, is less than 5%, at the rated speed of the turbine. This is based on a flow with a 1/7th power-law boundary layer profile and a MeyGen 1A turbine rotor, with a diameter of 18 m, rated speed of $3\;{\text{m s}}^{-1}$, hub height of 14 m and rotor drag coefficient of 0.8 [30].

From this, we conclude that the over-estimation in practical resource arising from neglecting support structure drag does not outweigh error sources that have caused the practical resource to be under-estimated.

Resource estimates from studies conducted since the Carbon Trust's assessment are compared in table 2, focusing on the Pentland Firth and Alderney Race as they exhibit the greatest tidal stream resource. Advancements have been made in the accuracy of hydrodynamic modelling through improved fidelity and temporal/spatial resolution, as well as improved validation, enabled through a greater availability of field measurements [34,35]. The most recent studies include at least eight tidal constituent forcings. All studies simulate the impacts of blockage caused by the turbine rotor drag, but exclude the contribution of support structure drag. The majority of the studies adopt a 2D (depth-averaged) modelling approach. Model validation demonstrates that these approaches are capable of capturing key tide-driven processes across the regional, array and turbine scales, and at acceptable computational cost [35]. Three-dimensional (3D) hydrostatic (layered) models, such as the ones implemented by O’Hara Murray, Dominicis and colleagues [20,32] are most useful in cases where high turbine density causes wakes to impinge on downstream turbines and/or in stratified flow, for example. They are significantly more computationally expensive, which can prohibit the number of model runs, if different array designs need to be considered, for example. Fully 3D, non-hydrostatic models are practically used for meso-scale and device-scale simulations that look to resolve fine-scale bathymetric features and individual turbines/turbine blades [36–38], but are currently too computationally expensive to cover the regional and array scales necessary to estimate the practical resource.

**Table 2.** Summary of time-averaged power estimates for the Pentland Firth, Scotland, and the Alderney Race, in the Channel Islands.

			constraints considered					
study	boundary forcings	2D/3D	Econ.	Env.	Reg.	Soc.	array layout and other considerations	time-averaged power
Pent. Firth								
Carbon Trust [16]	2	2D	✓	✓	✗	✗	n.a.	0.9 GW
Carbon Trust [16]	2	2D	✓	✓	✗	✗	environmental and economic constraints relaxed	2.0 GW
Adcock *et al.* [31]	2	2D	✗	✗	✗	✗	3 rows of turbines spanning the Pentland Firth, blockage ratio of 0.4	2.0 GW
Adcock *et al.* [31]	2	2D	✗	✗	✗	✗	1 row of turbines spanning the Pentland Firth, blockage ratio of 0.4	1.0 GW
Adcock *et al.* [31]	2	2D	✗	✗	✗	✗	1 row of turbines spanning the Pentland Firth, blockage ratio of 0.25	0.5 GW
O’Hara Murray & Gallego [32]	2	3D	✓	✓	✗	✗	1 row of turbines spanning the Pentland Firth, turbines occupy the bottom 25 m of the water column	1.4 GW
De Dominicis *et al.* [20]	8	3D	✗	✓	✗	✗	4.2 GW array spanning the Pentland Firth in waters deeper than 27.5 m	1.6 GW
Ald. Race								
Carbon Trust [16]	2	2D	✓	✓	✗	✗	environmental and economic constraints applied	0.18 GW
Carbon Trust [16]	2	2D	✓	✓	✗	✗	environmental and economic constraints relaxed	0.20 GW
Coles *et al.* [27]	1	2D	✗	✗	✗	✗	rows of turbines spanning entire width of Alderney Race, array density constrained to 0.038	1.40 GW
Coles *et al.* [28]	8	2D	✓	✗	✗	✓	2 GW array covering the majority of the Race, with a central channel left free for shipping	0.36 GW
Goss *et al.* [33]	9	2D	✓	✓	✗	✗	2.7 GW array, layout optimized for cost of energy, with constraints on array density	0.4 GW

As discussed, De Dominicis *et al.* [20] provide a promising insight into the economic and environmental viability of a large array in the Pentland Firth. However, all of the Pentland Firth studies simulate arrays that span the majority of the Channel width, thereby neglecting regulatory and social constraints that may limit the practical resource [20,31,32].

In the Alderney Race, economic, environmental and social constraints have been considered to an extent. Coles *et al.* [28] simulated an array that leaves a central channel for shipping, based on an array originally set out in [39]. The study also quantifies changes to the flow field as a result of blockage close to a large sandbank located south of Alderney [28]. Goss *et al.* [33] implemented optimization using gradient-based algorithms [40–42] to establish the footprint of arrays that minimize the cost of energy [33]. However, both studies acknowledge that sub-optimal rotor diameter and rated power of the turbines considered result in poor array performance, so further array design iteration is required. Studies investigating the environmental impacts of smaller arrays in the Pentland Firth and Alderney Race, with installed capacities an order of magnitude lower than the expected practical levels, are reviewed in §5.

Figure 3 shows that the Crown Estate Scotland has allocated 400 MW of lease capacity in the Pentland Firth at MeyGen and 40 MW around Islay (West Islay Tidal Project, 30 MW; Sound of Islay, 10 MW). The Crown Estate Scotland has withdrawn lease plots from its portfolio, such as the Ness of Duncansby, 100 MW; Brough Ness, 200 MW; and Brims, 200 MW, all located in the shallower regions of the Pentland Firth. In addition, the Westray lease plot in Orkney Waters (200 MW) and the Mull of Galloway lease plot on the west coast of Scotland (30 MW) have been withdrawn. At Holyhead, the Crown Estate has awarded a 10 MW agreement for lease at Holyhead Deep, and there are plans to build out the West Anglesey Tidal Demonstration Zone, also known as Morlais, currently up to 240 MW. In the Alderney Race, a total of 29 MW is being developed in its French territorial waters (Raz Blanchard) by Normandie Hydroliennes (12 MW) and Hydroquest (17 MW). There is currently a significant discrepancy between the required installed capacity to achieve 34 TWh/year of 11.5 GW and the 1 GW of allocated lease capacity currently available. This may not seem of immediate concern, given that increasing total installed capacity from its current level to 1 GW will take time. However, considerable spatial planning effort is required to establish if the required lease plot capacity can be allocated at each site/region. This will require joined up thinking between hydrodynamic modellers, sea-space commissioners, sea users and local communities.

Evidence has been provided that supports the 34 TWh/year practical resource estimate made by the Carbon Trust's study. However, the validity of the estimate relies partly on the accuracy of hydrodynamic modelling. We identify a need to update the national-scale practical resource estimate that has relied on models with generic site geometries, and limited validation data, with site-specific studies. The validity of the 34 TWh/year estimate also relies on the practical constraints neglected in the study having an insignificant impact on the practical resource, relative to the ones that have been implemented. This highlights a need to establish the sensitivity of the practical resource to individual constraints and to identify constraints that may reduce the practical resource and opportunities for retiring others. Research in this area can guide development of standards for resource assessment, such as IEC 62600-201 [43], in order to disseminate best practices and enhance the adoption of practical constraints in resource modelling.

## 3. Cost of energy

### (a) Levelized cost of energy

**Figure 5.**
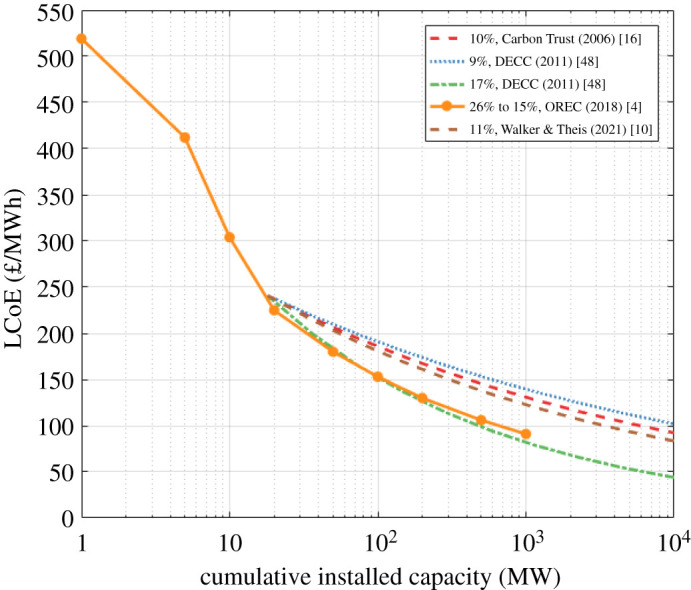
Relationship between cumulative installed capacity and LCoE, based on a range of technology learning rates reported in the literature.

LCoE is a metric commonly used to compare the economic performance of different energy projects [44]. It is the ratio of the total lifetime cost of a project to the energy output over its lifetime. LCoE projections are highly sensitive to capital and operational expenditure (referred to as CapEx and OpEx, respectively). Typically, future CapEx and OpEx are estimated using a technology learning rate, defined as the percentage reduction in costs with every doubling of cumulative installed capacity. While multiple factors will drive cost reduction, such as economies of scale and technology innovations (see appendix A for examples of these cost reduction drivers), the technology learning rate combines all cost reduction factors [45]. Typically, CapEx and OpEx projections adopt a wide range of technology learning rates of between 9% and 17% [10,46–49]. Cost data from operational projects demonstrate that, to date, tidal stream has achieved a technology learning rate of around 25% [4]. Future CapEx and OpEx projections also require an assumption to be made regarding the future cumulative installed capacity. The sensitivity of LCoE to technology learning rate and cumulative installed capacity is demonstrated in figure 5. LCoE data provided by OREC are based on information provided directly by tidal stream project developers [4]. Based on these data, the learning rate starts at 26% during the early phases of development, then reduces to around 15% after cumulative installed capacity exceeds 100 MW. Other technology learning rates range between 9% and 17%, resulting in significantly different future LCoE projections, ranging between 80 and 140 £/MWh after 1 GW of cumulative installed capacity, for example.

**Figure 6.**
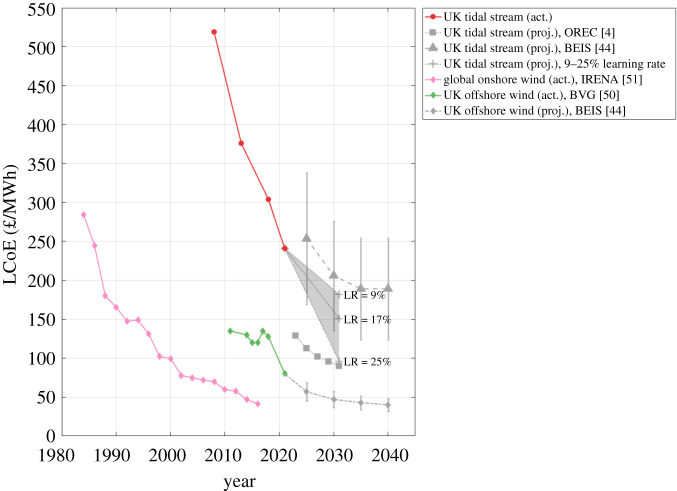
LCoE of tidal stream [4,44], UK fixed-bed offshore wind [50] and global onshore wind [51], based on actual data from operational projects and projections. Tidal stream LCoE projections are given based on learning rates (LR) ranging between 9% and 25%.

Figure 6 compares actual LCoE data from operational projects with LCoE projections provided by OREC [4] and BEIS [44]. The LCoE values of global onshore wind [51] and UK fixed-bed offshore wind [50] are also plotted for comparison. Tidal stream is on a steep cost reduction trajectory, where the installation of the first 8 MW of tidal stream capacity in the UK led to a reduction in the LCoE of approximately 25%, to around 300 £/MWh [4]. This is similar to the cost reduction rate achieved by onshore wind between 1985 and 1990 [51].

We find that the OREC projections are likely to under-estimate future LCoE. The projections assume that cumulative installed capacity increases at a rate of 100 MW/year, from 2021/22. This projected build-out rate is shown in figure 1, and is now acknowledged to be unachievable if projects are to rely on CfD subsidy support, which may only facilitate this level of build-out to commence around 2026. A counter argument to this is that OREC’s LCoE projections are based on aggregated data from multiple tidal stream turbine developers, with devices ranging from the kW to MW scale [4]. The LCoE of larger devices is likely to fall below the aggregated projection as a result of economies of scale, as described in appendix Aa.

BEIS provides ‘high’ and ‘low’ LCoE projections, based on a pessimistic to optimistic range of CapEx and OpEx inputs, respectively. Error bars in figure 6 indicate these high and low LCoE projections. BEIS uses a high CapEx for an 18 MW array installed in 2025 of approximately 8.1 m£/MW, showing close agreement with MeyGen 1A’s reported CapEx of 8.6 m£/MW [19]. BEIS uses a high OpEx cost of approximately 0.7 m£/year, only half the reported OpEx of MeyGen 1A [19]. Construction costs, which contribute to CapEx, are subject to a learning rate that is equivalent to a cost reduction of 1%/year [48]. This cost reduction has been implemented up to 2030, with no further increase in cumulative installed capacity after 2030. LCoE projections are all based on an 18 MW array. These assumptions regarding increases to cumulative installed capacity, and individual array scale, neglect the full impacts of economies of volume, and learning, on LCoE. The BEIS 2025 high projections lie above the reported 2018 LCoE of tidal stream from operational projects, of 304 £/MWh [4]. It is therefore likely that neglected cost reductions from learning and economies of volume outweigh the cost reductions caused by under-estimating CapEx and OpEx.

Figure 6 also provides three 2031 LCoE projections, based on findings from this review. In all three cases, the 2031 cumulative installed capacity is estimated to be 160 MW. The first contribution to this is the UK's current cumulative installed capacity of 18 MW. The second is 124 MW of additional capacity installed in the UK at sites that are currently eligible to bid for CfD support: Morlais (14 MW), PTEC (30 MW) and MeyGen 1C (80 MW). The third is an additional 18 MW installed outside the UK, derived by linearly extrapolating the global cumulative installed capacity (excluding the UK) achieved to date, which is shown in figure 1. A 2031 installed capacity of 142 MW in the UK and British Channel Islands is necessary to put it on a trajectory to achieve its practical resource potential of 11.5 GW by 2050, as illustrated in figure 1. The LCoE projections adopt a technology learning rate of 9%, 17% and 25%, reflecting the extreme and mid-range estimates in the literature and the technology learning rate achieved to date from operational projects. These yield 2031 LCoE estimates of 182 £/MWh, 150 £/MWh and 96 £/MWh, respectively. The mid-range LCoE estimate of 150 £/MWh by 2031 falls between the BEIS and OREC LCoE projections, as expected for the reasons discussed. A technology learning rate of 17% also achieves an LCoE reduction trajectory that agrees closely with OREC's, as shown in Figure 5. OREC's LCoE projection is based on cost data obtained from turbine developers, and results in an LCoE projection of 90 £/MWh after 1 GW of cumulative installed capacity [4]. Given the high sensitivity of LCoE to the technology learning rate, it will be important to monitor its progression with cumulative installed capacity in the future, to update LCoE projections if necessary.

### (b) Cost competitiveness

**Figure 7.**
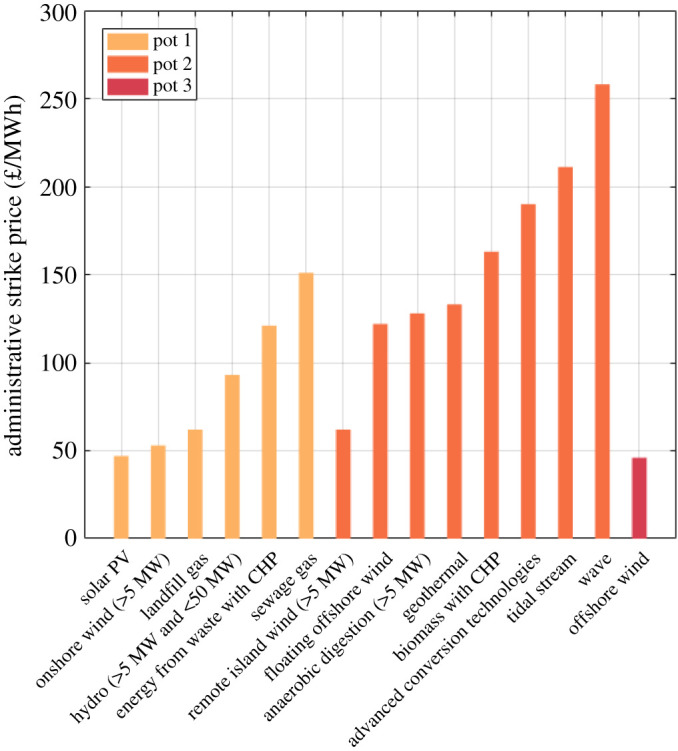
Comparison of AR4 technology administrative strike prices, based on 2011/12 prices [15].

In September 2021, BEIS announced that, in AR4, tidal stream energy projects will be eligible to compete for CfD subsidy support in ‘pot 2’ [15]. As well as tidal stream, pot 2 includes advanced conversion technologies (such as gasification), dedicated biomass with combined heat and power (CHP), floating offshore wind, geothermal, remote island wind (greater than 5 MW) and wave. Pot 2 has a budget of 55 m£/year, with 24 m£/year ring-fenced for floating offshore wind only. All budgets are based on 2011/12 prices. Consideration by BEIS for ring-fencing of other technologies is ongoing, and will be decided before the AR starts in December 2021. The administrative strike price, which defines the minimum strike price projects of a particular technology can bid for, is shown for each technology in figure 7. Administrative strike price is only indicative of the cost competitiveness of different technologies. However, given the vast difference in administrative strike price between tidal stream, of 211 £/MWh, and competing technologies such as remote island wind, of 62 £/MWh, it is unlikely that tidal stream will be able to win subsidy support in AR4, unless a ring-fence is introduced for it.

**Figure 8.**
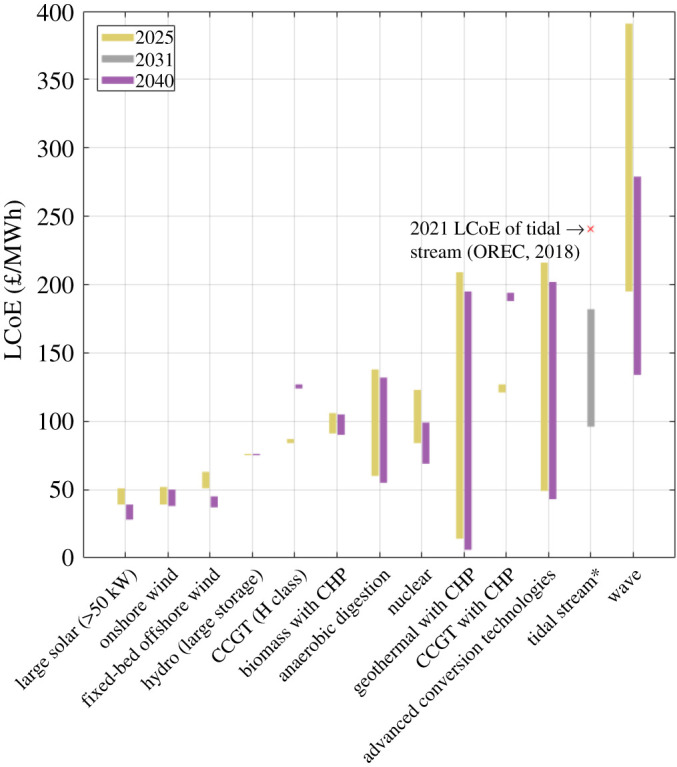
Comparison of 2025, 2031 and/or 2040 LCoE projections for different technologies [44], based on 2011/12 prices. *Tidal stream projection based on findings presented in this review.

Figure 8 provides LCoE projections for a wide range of power generation technologies. With the exception of tidal stream, data are based on 2025 and 2040 LCoE projections provided by BEIS [44]. The 2031 tidal stream LCoE projection is based on previously stated conclusions, which support a mid-range LCoE of 150 £/MWh, and lower and upper bounds of 96 £/MWh and 182 £/MWh, respectively. The current LCoE of tidal stream is also shown [4]. The projections indicate that, by 2031, the LCoE of tidal stream has the potential to become competitive with that of combined cycle gas turbines (CCGTs) (both H class and with CHP), biomass with CHP, anaerobic digestion, geothermal with CHP and advanced conversion technologies.

Figure 8 shows that the estimated cost reduction trajectory of tidal stream is steeper than the majority of other technologies, including those in AR4 pot 2. For the LCoE of tidal stream to become competitive with the 2040 LCoE of nuclear, of around 90 £/MWh), the global cumulative installed capacity of tidal stream must reach approximately 1 GW, based on a technology learning rate of 17%. This is equivalent to 9% of the estimated total installed capacity required to generate the UK practical resource, of 11.5 GW, suggesting significant further cost reduction is likely with additional tidal stream installations after 1 GW. These two findings support the argument for a ring-fence to be introduced for tidal stream in AR4.

Developed technologies such as wind and solar are on a flatter cost reduction trajectory to 2040, having already reduced cost through their earlier adoption. The LCoE of CCGT technologies (both H class and CHP mode) is projected to increase to levels exceeding 120 £/MWh by 2040. Currently, the UK has approximately 32 GW of CCGT capacity that supplies over 25% of the UK’s annual electricity demand. Importantly, CCGT is capable of providing dispatchable generation in periods of low renewable energy resource, so it is likely to play an important role in the coming years as more variable renewable capacity is connected to the grid. The same may also be true of biomass, which currently provides around 11% of the UK’s electricity demand, but at much higher projected cost that is not expected to reduce significantly between 2025 and 2040.

## 4. System integration

### (a) Balancing

In the 2019 offshore wind sector deal, the UK government identified the grid integration of variable generation as a key challenge for the industry as renewable power penetration increases [52]. Balancing between supply and demand is central to this challenge. Resource availability defines the percentage of time a generation resource is available for supplying demand. In comparison with other variable generators, tidal stream has been shown to exhibit relatively high resource availability [53], in part because of the cyclic nature of the tides [54]. These characteristics of tidal power generation are described here.

**Figure 9.**
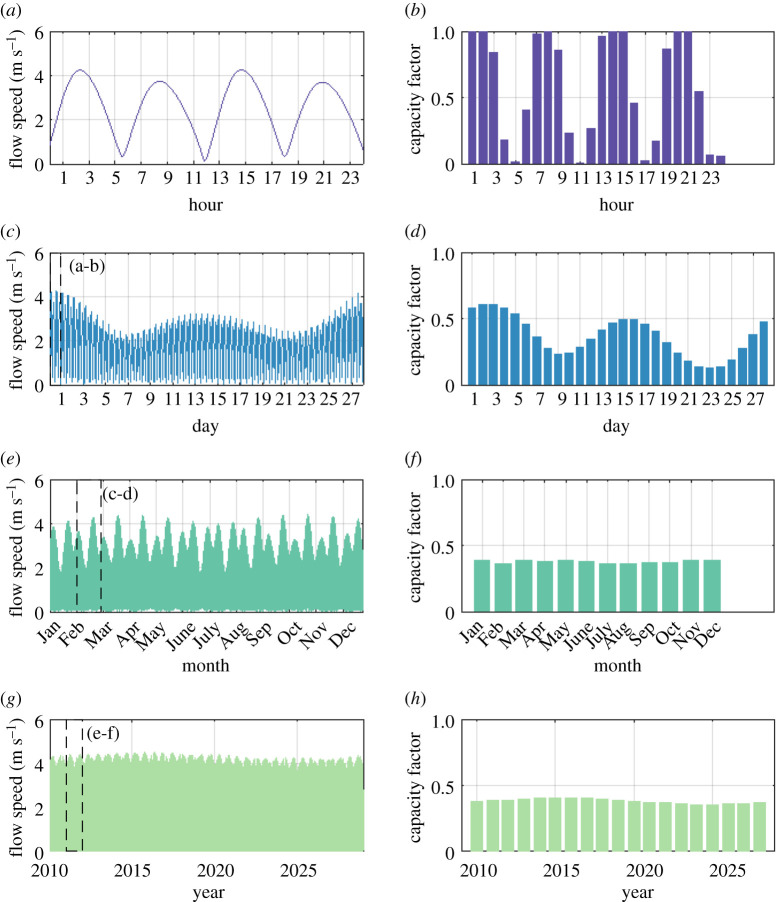
Demonstration of the cyclic, predictable nature of tidal flow and generated power, over time scales of (*a*,*b*) a single day, (*c*,*d*) a month, (*e*,*f* ) a year and (*g*,*h*) 19 years.

The UK has semi-diurnal tides with a period slightly greater than 12 h [55]. In this period, the tides complete a flood–ebb cycle, with slack water separating flood and ebb tides. Tidal stream power generation occurs in periods when the cut-in speed of the turbine is exceeded, and ceases during slack water. The resulting power signal has approximately four periods of generation per day, every day. This semi-diurnal cycle of flow speeds is shown in figure 9*a*. Figure 9*b* provides the typical hourly capacity factor of a tidal stream turbine over the same time period, and assumes 100% turbine availability and no electrical losses between the turbine and the grid. Power generation is greatest during spring tides, when alignment between the Sun, Earth and Moon, known as syzergy, maximizes the tide generating force. Neap tides exhibit lower flow speeds, and therefore power, owing to mis-alignment between the Sun, Earth and Moon. These daily variations in flow speed and capacity factor are shown in figure 9*c*,*d,* respectively. The variation in flow speed caused by the spring neap cycles over a year are shown in figure 9*e*. Since the distribution of spring and neap tides is similar over each month, monthly capacity factors remain fairly consistent at around 0.4, as shown in figure 9*f*. At longer time scales, the tides are affected by the 18.6 year lunar nodal cycle, which is mainly driven by variation in the inclination of the Moon's orbital path relative to the equatorial plane of the Earth. The annual capacity factor of turbines (figure 9*h*) shows approximately a ±10% variation over this period.

By contrast, variations in the monthly capacity factor of the UK’s wind energy fleet can be significant. On average, the monthly inclusive capacity factor of wind power is approximately 0.3, but over June 2014 it dropped to 0.11 [56] as a result of a large-scale weather system [57]. A similar drop in capacity factor from the UK’s proposed 40 GW offshore wind power capacity in 2030 [58] would result in a reduction in power of approximately 7.6 GW, equivalent to approximately 20% of the UK’s current time-averaged electrical power demand. When the available wind power exceeds the grid capacity, power is typically curtailed. Between 2015 and 2020, curtailment of UK wind energy has increased from 1.22 TW/year to 3.82 TWh/year, rising approximately in line with annual wind energy generation. The cost implications of the impacts of variability on balancing are reviewed in §4c.

The predictability of the tides is also an important advantage for system balancing. With knowledge of astronomical cycles, tidal elevations and velocities, and therefore power, can be predicted at sub-hourly resolution centuries into the future using numerical models and harmonic analysis [55,59,60]. This high certainty over future generation can reduce the level of intervention required to achieve system balancing, relative to more unpredictable technologies [61]. In contrast to tidal stream energy, other variable power generation technologies tend to exhibit relatively high levels of forecasting uncertainty, which can lead to power balancing shortfalls [53]. A study of Spanish wind power forecasts shows that the mean absolute error in average production reduced from approximately 15% 2 days in advance to around 6% 1 h in advance [62]. At high penetrations, the magnitude of the uncertainty in instantaneous power generation will be significant, requiring larger, more rapid and more costly interventions to balance and stabilize the system [63–68]. These costs are discussed in §4c.

### (b) Grid connection and storage

Regions of highest tidal stream resource in the UK are located in remote areas, where electricity demand is low and access to the transmission grid is limited. Regional grid constraints are illustrated in figure 3, through mapping of grid boundary capacity (GBC). GBC is defined as the net power transfer limit between regions [69]. For the UK and British Channel Islands to meet the current practical resource estimate of 34 TWh/year, it is estimated that 6 GW and 2 GW of tidal stream capacity must be installed in the Pentland Firth and British Channel Islands, respectively. Both regions also exhibit high wind resources that have started to be developed, with future development planned, making the regions net exporters of renewable power. The Pentland Firth and Channel Islands currently have grid boundary capacities of 1 GW and 0 GW, respectively, limiting/preventing any future large tidal stream power from being transmitted to high-demand centres. Grid studies have found that sites in North Scotland will require significant reinforcement with long development time frames [70].

Wales and the Bristol Channel region have relatively high grid boundary capacities, of 10.3 GW and 2.1 GW, respectively. High-demand centres such as Swansea, Cardiff and Bristol are in relatively close proximity to the tidal stream resource in the Bristol Channel. It is expected that tidal stream generation at sites such as Portland Bill and those in the Bristol Channel will be able to connect into the transmission and distribution network close to the shore without significant grid reinforcement [70]. Other sites on the south coast of England and in Wales are likely to require some grid reinforcements back to inshore substations or the transmission network, and/or longer sub-sea cables. It is estimated that the grid around the North Irish Sea and North Channel is capable of exporting around 20 MW, but higher export power will require significant onshore reinforcements and long sub-sea cables.

The cyclic nature of tidal power production is well suited for integration with short-term energy storage (less than 4 h) to help balance supply with local demand [54,71–74]. During spring tides, excess tidal power is used to charge the battery. During slack water periods, the battery discharges to meet demand. The cycle then repeats. Periods of no tidal power generation (i.e. slack water) last for approximately 2–3 h, so energy storage systems with the same storage duration allow power to be supplied during these low tidal resource periods. This can be achieved using lithium-ion batteries [75] or flow batteries [76]. During neap tides, reliance on back-up power increases. This type of embedded generation can prevent the need to reinforce connections with the transmission grid.

Commercial tidal stream projects have now started adopting energy storage. The 0.4 MW Shetland tidal stream turbine array has been connected to lithium-ion batteries to provide continuous power [75]. The Orbital Marine Power (previously Scotrenewables) SR2000 and Tocardo TFS and T2 tidal turbines have been used to generate hydrogen at EMEC [77]. EMEC has also announced that it will combine tidal stream power with a 1.8 MWh flow battery to power its hydrogen production plant [76].

### (c) Whole-system costs

Conventionally, LCoE does not account for the whole-system costs incurred by different generation technologies as a result of balancing, grid reinforcement and transmission [62,63]. This is consistent with the data presented in §3. Whole-system costs are defined as the change in costs that are incurred from constructing and operating the power system with the addition of a new plant [63]. The whole-system cost will depend on the prevalence of complementary technologies, forecast accuracy and the size of variable plant considered relative to the transmission capacity. ‘Enhanced’ LCoE (eLCoE) accounts for these whole-system costs to provide a more well-rounded representation of cost of energy. BEIS estimates that the eLCoE of UK fixed-bed offshore wind in 2035 will be between 60 and 80 £/MWh. This is 50–100% higher than its projected 2035 LCoE, of 40 £/MWh [44].

While the eLCoE of tidal stream remains unclear, initial studies show that diversifying a 100% wind portfolio that generates 120 TWh/year, by replacing 25% of its energy production with an even split of tidal stream and wave energy, reduces balancing expenditure by approximately 700 m£/year [78]. These cost savings are achieved by reducing back-up capacity, reduced costs of reserve capacity and reduced fuel costs. This cost saving is equivalent to approximately 3% of the annual wholesale cost of electricity. Given this potentially high cost saving, further work is required to strengthen these findings, by quantifying the whole-system cost and eLCoE of tidal stream, to compare against competing technologies, using accepted approaches such as those set out in [79].

## 5. Environmental impact

### (a) Hydro and sediment dynamics

Large-scale energy extraction by tidal stream turbine arrays modifies the surrounding ambient flow field as a result of the added turbine drag. In general, tidal stream turbine arrays cause an increase in upstream tidal elevation, and a downstream decrease, typically of a few centimetres [27,80]. Flow speeds are seen to increase around arrays, and reduce in the array wake [28]. Flow speed reduction can decrease the energy of tidal mixing, perturbing the balance between stratification and vertical mixing processes. Importantly, the impact of large arrays on stratification has been shown to be an order of magnitude lower than those caused by climate change [20].

O’Hara Murray & Gallego [32] investigated far-field impacts from energy extraction in the Pentland Firth by simulating a row of turbines spanning the width of the Firth, covering the lower 25 m of the water column, and generating a time-averaged power of 1.4 GW [32]. It was estimated that the array would reduce the time-averaged volume flux through the Pentland Firth by 7%. Flow speeds in the Firth reduced by around $0.5\;{\text{m s}}^{-1}$, and the tidal range within the Pentland Firth and Scapa Flow was modified by 0.1 m.

The transport of sediment is approximately related to current speed cubed; therefore even a modest change in the velocity field from turbine installations can lead to a significant impact on sediment dynamics [81]. This will depend on the number of turbines, their proximity to sedimentary deposits and site-specific characteristics such as the hydrodynamics and the sediment type/amount/distribution. Near-field impacts include scour, which may affect array operation, while far-field effects could have an impact upon the structure of larger features such as sandbanks. Typically, high-energy tidal stream sites are characterized by low levels of sediment, as the ambient flows winnow finer sediment, leaving coarser sediment and bedrock. However, sediment can accumulate in lower flow regions of high-energy sites, as is seen at MeyGen [82,83] and the Alderney Race [84,85], as well as headland sites associated with energetic tidal flows, such as Portland Bill [86].

A 2015 study investigated the cumulative effect of MeyGen (86 MW), Ness of Duncansby (95 MW), Bough Ness (99 MW) and Brims array (200 MW), in the Pentland Firth, on sediment transport [87]. Positively, results indicate that the arrays cause minimal modification to the baseline morphodynamics of neighbouring large sandbanks, suggesting changes to the ambient flow field are unlikely to cause detrimental impact to sediment morphology. Results from modelling of energy extraction around Holyhead, Wales, indicate that arrays with an installed capacity of up to 50 MW reduce flow speeds locally by a few per cent [88]. This change is negligible relative to the natural flow variability. Model predictions show that when the array capacity increased to over 50 MW sedimentary processes were significantly affected, but that energy extraction was unlikely to alter bed shear levels past their natural levels of variability further afield (10 km away from the array). A similar study at MeyGen found that changes to natural patterns in sediment migration are possible once the array capacity exceeds approximately 85 MW, where flow diversion around the array may cause long-term accumulation of coarse sediment and gravel in the centre of the array and scouring and removal of existing sediment deposits to the north and south of the array [89]. These findings are consistent with those in other studies [90,91]. Another study looked at the impact of 300 MW arrays on sediment transport around the Alderney South Banks, a large sand bank to the south of Alderney in the Alderney Race caused by a large eddy system [85,92]. Results show that most of the high-energy array locations considered would be unlikely to affect the flow field in the vicinity of the South Banks; however, in some cases, the array caused asymmetrical modifications to the flow and sedimentary regime. These findings must acknowledge that numerical modelling of sediment dynamics is highly challenging, in part because of the large range of temporal and spatial scales involved [85], as well as uncertainties in aspects such as the suspended and bedload sediment supply in and out of the regions modelled [89].

In summary, hydro and sediment-dynamic modelling to date highlights that, as arrays scale up in size, they have the potential to modify the surrounding flow field and sediment transport. Given the relatively high level of uncertainty in sediment transport models, there is a need for complementary field measurement campaigns that track the changes that arrays of increasing size make to surrounding sediment dynamics, as the industry develops. Impacts on sediment transport are closely linked to the proximity of arrays to sedimentary deposits. Clearly, this consideration should be prioritized in the spatial planning of large arrays to help mitigate detrimental impacts.

### (b) Collision risk

There is a longstanding concern that marine mammals, fish and diving seabirds could be injured or killed as a result of collisions with the rotating blades of tidal turbines, in a similar fashion to birds colliding with wind turbines [93–95]. In general, for a small number of marine mammal species, monitoring around single turbines and small arrays has provided evidence of avoidance in the range of hundreds to thousands of metres, which would lead to lower estimates of collision risk compared with worst-case assumptions [96–100]. A recent study provided the first evidence for fine-scale evasion of an operational tidal turbine by harbour porpoises [101].

Monitoring has provided evidence that some species of fish aggregate around turbines during periods with low current speeds, possibly to use the structure for shelter from the flow or for feeding strategies [102]. Other studies have demonstrated avoidance and individual evasion around rotating river turbines and have concluded that collisions are absent or infrequent [103–105].

Less is known about the risk of collision between tidal turbines and diving seabirds [106]. Nova Innovation has collected 20 000 h of video data over 5 years from its array in Bluemull Sound, Shetland, and 20% of this footage has been examined. Underwater video collected and sampled recorded black guillemot and European shag close to the turbines. All observations were during slack tide or at flow speeds that were too low for turbines to generate power. Shags were observed actively pursuing fish around turbines but no physical contact between birds and turbines was observed. The data included less than 30 marine mammal/bird sightings in close proximity to the turbines, and no evidence of collisions [107].

A recent programme of research, funded by the Scottish government, was carried out at MeyGen 1A in the Inner Sound (Pentland Firth) to provide a better understanding of collision risk posed by tidal turbines [101]. The full suite of outputs from this work is not yet published but initial results from the research show the following.

— Harbour seals foraged in tidal development sites but spent very little time within 100 m of operating tidal turbines.
— Seals avoided the turbines while they were operating.
— Fine-scale tracking of harbour porpoises in the vicinity of the operational turbine revealed evidence of localized evasion; over 451 days of monitoring, no tracks were detected going through the rotor swept area while the turbine was operating with only a single tracked animal passing through the rotor area while the rotors were stationary.
— Porpoises frequently swim within 150 m of operational tidal turbines, but occurrence is between 33% and 78% lower during periods of turbine operation, relative to non-operation.
— Dolphins were detected by hydrophones, mostly in winter (which is similar to porpoises), but at a much lower rate.
— Fish schools were at much higher abundances during neap versus spring tides. There is most likely a threshold effect between the amount of fish available and the number of foraging seabirds [108].
— Seabird species will be affected differently by the presence of tidal turbines as pelagic-foraging species detections were found to be related to dynamic fish school locations, whereas benthic-foraging species detections were linked to set locations in the site [108].

It is important to also highlight that, while the monitoring techniques used to date are designed to detect collisions, none has the capacity to reliably determine whether a collision has occurred. Information on the fine-scale underwater movements (at a scale of metres) of individual animals of a range of species across different taxa (other mammals, birds and fish) around operating turbines remains a critical research gap with respect to understanding the potential impacts of tidal devices.

To conclude, good progress has been made to improve understanding of how marine mammal and fish species respond to operating turbines at a range of spatial scales. There is currently no evidence of collisions between turbines and protected marine animals. In the Pentland Firth, it was shown in §2 that relatively low array density is required to achieve the 6 GW installed capacity necessary to extract its practical resource estimate. In practice, as arrays increase in size, turbines will be distributed non-uniformly, resulting in areas of higher collision risk than others. Monitoring will probably be needed throughout the array expansion process to improve understanding of how collision risk scales with array size and spatial distribution of turbines. The focus of monitoring will probably require a shift from understanding how individuals behave in the immediate vicinity of single turbines to how individuals behave between devices in an array.

### (c) Habitat change and displacement

Evidence to date suggests that habitat displacement caused by single devices and small arrays is relatively small scale. For example, studies of harbour seals have demonstrated empirical evidence for displacement of between a few hundred metres [97,98], to a maximum of 2 km [100]. These represent small-scale responses relative to the scale of movements generally exhibited by these species. There are fewer studies on cetacean species but there is evidence of a local-scale reduction in activity at the scale of tens to a few hundred metres around devices [99]. Although apparently relatively minor, the significance of this displacement may depend on the location and the availability of alternative habitat. Seabird data collected from operational wind farms show that, when animals are displaced from historical feeding areas, local abundance levels can be affected significantly [109].

Another potential cause of displacement is the change to the physical environment (i.e. the habitats of foraging mobile animals) in locations of energy extraction and downstream of an array [20]. However, modelling studies indicate that these changes are likely to be relatively small compared with the impacts of climate change and the effects this will have on how animals are going to change the way they feed [110]. Similarly, the effects of energy extraction on predator–prey relationships are expected to be small relative to the impacts of climate change [80].

Published studies on the effects on benthic habitats and species are relatively rare, possibly reflecting regulatory priorities that often focus on the impacts of protected pelagic species. Additionally, long observation periods are required to detect long-term changes in such habitats, which can be challenging to obtain. In addition to the direct habitat loss that results from the footprint of seabed-mounted devices and from associated cables, benthic habitats can also be altered by local changes in turbulence and the creation of new habitat for colonization; however, these changes have been demonstrated to be very localized [111,112]. Modelling studies have also predicted that any changes to biomass are likely to occur within the area of developments rather than outside of them [113].

The 2020 State of the Science report [114] concludes that tidal stream turbines may provide habitats for biofouling organisms, while also attracting fish and other animals, through the creation of artificial reefs. This has the potential to alter fish populations in surrounding areas. Overall, changes to habitat are likely to pose a low risk to animals, if turbine deployment in fragile or sensitive habitats is avoided. Long-term studies of changes to habitats will be required to understand whether there is the potential for such changes to result in any ecological significance and to validate predictive models.

Noise is produced at all stages of a tidal turbine project, from construction to operation and decommissioning, with the potential to affect the surrounding ecosystem, from primary producers to top predators. Of the single devices that have been measured to date, the potential for auditory injury and habitat displacement appears to be low for marine mammals and fish [115]. Measurements taken within 100 m of the turbine during low, neap, sea states show that noise levels are elevated by approximately 30–40 dB as a result of the turbine’s noise emissions. This is equivalent to an increase in noise levels of 30–40%, based on ambient noise measurements of 100 dB. The level of turbine noise elevation reduced to 5 dB at a distance of 2.3 km away from the turbine [116]. Ambient noise during spring tides will be considerably higher than that recorded during the experiments. On a small scale, it has been shown that harbour seals avoid simulated tidal turbine sounds [98] and harbour porpoise click activity was significantly reduced compared with baseline levels within a few hundred metres of an active device [99].

Based on these findings, it is concluded that the risk of habitat displacement from single devices/small arrays is relatively low. Continued monitoring of animals at operational tidal stream energy projects is required to establish the impacts of larger arrays as they scale up. Methods of measurement must adhere to those set out in technical standards, such as the International Electrochemical Commission technical standard 62600-40:2019—Part 40, which is becoming the accepted means of measuring acoustic output from tidal turbines [117].

## 6. Conclusion and recommendations for future research

To achieve net-zero targets, a diverse fleet of cost-effective renewable power generation technologies are needed. The latest tidal stream practical resource estimate of 34 TWh/year is equivalent to 11% of the UK’s current annual electricity demand. Evidence has been provided that helps validate the estimate, in terms of both the method used to estimate energy extraction and the economic and environmental constraints implemented that cap the resource to practically achievable levels.

We estimate that, for 34 TWh/year to be achieved, approximately 11.5 GW of installed capacity is required, with 6 GW in the Pentland Firth, Scotland, and 2 GW in the Channel Islands. Significant grid reinforcements would be required in the two regions to transmit power to high-demand centres. Sites located on the south coast of England and in the Bristol Channel have relatively good access to local grid infrastructure, thus helping to limit whole-system costs.

We show that the LCoE of tidal stream is on a steep downward trajectory relative to other technologies. LCoE is intrinsically linked to cumulative installed capacity, with future installations reliant on government subsidy to provide a route to market. In the UK, three projects are eligible to bid for CfD subsidy support in AR4 under current rules (Morlais, 14 MW; PTEC, 30 MW; and MeyGen 1C, 80 MW). We estimate that the build out of all three projects, alongside 18 MW globally, can reduce LCoE from its current level of approximately 240 £/MWh to less than 150 £/MWh, making tidal stream cost competitive with technologies such as CCGTs, biomass and anaerobic digestion. This projection is based on a technology learning rate of 17%, informed by future array costs, which is conservative relative to the 25% technology learning rate achieved by operational projects to date. In recent years the rate of installed capacity increase in the UK has slowed, owing to the lack of subsidy support. Installing 124 MW at MeyGen 1C, PTEC and Morlais by 2031 would put the tidal stream industry back on a trajectory to install its 11.5 GW potential by 2050.

It has recently been announced that, in AR4, tidal stream will compete for subsidy support in pot 2 against technologies such as remote island wind and floating offshore wind. The majority of the pot 2 technologies have a significantly lower administrative strike price than tidal stream. This makes it unlikely that tidal stream will be able to secure subsidy support, unless a ring-fence is provided to give subsidy access that is uncontested by other technologies, similar to the one being provided for floating offshore wind.

The cyclic, predictable nature of tidal stream power generation has been shown to deliver whole-system cost savings that can improve its cost competitiveness relative to other variable generation technologies. We identify this to be a key area for further research, given that cyclic and predictable power generation are two widely assumed benefits of tidal stream that are only supported through initial estimates of whole-system cost savings. This is particularly pertinent given that the penetration of variable power sources onto the grid is increasing, providing significant grid integration challenges, such as supply–demand balancing.

We find no evidence that tidal stream turbines have caused significant detrimental environmental impacts to date. Environmental monitoring of single/small arrays of turbines has improved understanding of collision risk between turbines and animals. Similarly, evidence shows that single devices and small arrays have relatively small-scale impacts on sediment distribution and habitat displacement. It is important to contextualize environmental impacts, where, for example, changes to stratification and predator–prey interaction, caused by tidal stream development, are projected to be an order of magnitude less than those from climate change. Ongoing field measurements are required to establish the impacts of array scale on environmental impacts as the industry develops, since in many areas modelling is not yet capable of providing the necessary accuracy required.

The evidence presented in this review broadly supports the latest UK practical resource estimate of 34 TWh/year. To reduce uncertainty in the estimate further, we recommend that an updated national-scale practical resource study is conducted that implements (i) improved fidelity, site-specific hydrodynamic modelling, (ii) enhanced model validation, which uses all relevant field measurements taken at each site, (iii) sensitivity analysis to investigate the impacts of economic, environmental, regulatory and social constraints on the practical resource, and (iv) quantification of whole-system costs and eLCoE, relative to competing technologies.

## Data accessibility

This article has no additional data.

## Appendix A. Cost-reduction mechanisms

### (a) Economies of scale

Economies of scale relate to the size/specification of infrastructure installed on a project. When applied to the turbines themselves (e.g. an increase in the rotor diameter), this can result in significant increases in energy production per turbine. This reduces the number of turbine installations required to meet a given array capacity, leading to a reduction in the number of installation, maintenance and decommissioning operations, as well as minimizing other infrastructure costs such as cabling [19]. Any cost benefit from economies of scale must exceed any additional cost incurred from increased turbine loading, for example [118]. The diameter of the Orbital Marine Power rotors has increased from 16 m on the SR2000 to 20 m on their next iteration, the O2. This has also led to a reduction in hub height of approximately 2 m, since both the SR2000 and O2 are floating devices. The increased diameter of the rotors increased the total swept area of the device by 56%. When the device is operating at below rated speed, power generation is directly proportional to swept area, so similar levels of power generation increase are expected. A new tidal stream turbine cost model has been developed that estimates that this increase in rotor diameter is achievable with an increase in turbine CapEx of 41% [119]. This demonstrates that the uplift in energy yield from increases in turbine scale can outweigh the additional CapEx incurred, and that significant increases in turbine scale are achievable within the time frame of LCoE projections presented in §3.

Table 3 summarizes economies of turbine scale and their impact on energy yield. Combining these economies of scale can result in significant increases in annual energy yield per turbine. For example, a 3 MW, 24 m turbine with a 17 m hub height can produce nearly double the annual energy of a 1.5 MW, 18 m turbine with a 14 m hub height [120].

**Table 3.** Summary of economies of scale.

mechanism	description
rotor diameter	there has been a gradual increase in rotor diameter from *ca* 5 m in 1990 to 20 m on current turbines. Increases in power generation scale approximately with the rotor radius squared. It is estimated that increasing the rotor diameter of the current MeyGen phase 1A turbines from 18 m to 24 m would increase energy yield by 34% [120].
rated power	the rated power of turbines has increased from *ca* 15 kW in the 1990s to 2 MW on devices deployed in 2021. It is estimated that increasing the rated power of a 24 m rotor diameter turbine at MeyGen from 1.5 MW to 2 MW increases annual energy yield per turbine by 17% [120].
hub height	based on the typical vertical boundary layer profiles at tidal stream energy sites, approximately 75% of the energy is in the upper 50% of the water column. Floating devices position their rotors in this higher energy region of the water column, enabling energy yield to be maximized for a given location. It is estimated that energy yield increases by approximately 2% per metre increase in hub height [120].

### (b) Economies of volume

Experience from the offshore wind industry suggests that, as the number of turbines within arrays increases, common components become standardized, enabling production costs per unit to be reduced through mass manufacturing [4]. Lessons from the MeyGen 1A project highlight that the four-turbine array is not enough to drive unit cost reductions because dedicated production facilities and tooling could not be justified, but that larger turbine volumes are expected to bring these economies of volume in the future [19]. Increasing turbine volume allows some costs to be shared across turbines. For example, a significant cost related to the installation of large seabed-mounted turbines is the mobilization and de-mobilization of dynamic positioning vessels, where the vessel is loaded with all necessary equipment and infrastructure and unloaded after use. The costs of mobilization/de-mobilization per turbine reduce as the number of turbines to be installed increases. It has been estimated that a 36 per turbine array reduces mobilization and de-mobilization days per turbine by 26% relative to a four per turbine array, with a similar level of vessel cost saving expected [120].

### (c) Technology innovation

Table 4 summarizes examples of technology development that show promise to drive down costs.

**Table 4.** Summary of technology innovations.

mechanism	description
sub-sea hub	sub-sea hubs provide a central point to join multiple turbines to a single export cable. The sub-sea hub reduces the number of export cables and onshore power converters, while also reducing cable installation time per turbine. There is an estimated 80% saving in the total cost of the associated infrastructure (i.e. cables, converters etc.) as a result of using sub-sea hubs [120].
cable design	cable incidents cause 85% of the insurance claims related to offshore wind. For fixed tidal stream systems, cables are estimated to represent 14% of total CapEx. Recent research demonstrates that rocky seabeds have a wide fluid boundary layer and high seabed friction, owing to the ruggedness. The observed stability of the power export cables used on the MeyGen 1A tidal stream project, which could not be certified as stable using the conventional design approach, supports this research [121,122]. By redesigning cables accordingly, cost reductions will emerge.
wet-mate connectors	the connection between the export cable and the turbine is made using either dry-mate or wet-mate connectors. Dry-mate connectors must make the cable–turbine connection out of the water, which is done during turbine installation/retrieval by lifting the export cable from the seabed to the deck of the vessel. Wet-mate connectors allow the turbine–cable connection to be made sub-sea, allowing the export cable to remain on the seabed. By avoiding the need to manipulate the export cable, wet-mate connectors simplify offshore operations, reducing the time needed to carry out a turbine installation/retrieval, thereby reducing the overall installation cost by around 65% relative to turbines using dry-mate connectors. Wet-mate connectors also de-risk cable damage by reducing the number of times the cable is moved over its life [19].
foundations	seabed-mounted tidal stream turbines use heavy gravity-base foundations (GBF) to maintain stability. The cost of GBFs is approximately 11% of the CapEx [19,118]. It is estimated that the adoption of monopile foundations can reduce the amount of steel used per foundation by 90%, which can lead to cost savings. Alternative emergent technologies such as remotely operated micro-pile installation rigs, as well as the potential to unlock higher seabed friction for a conventional gravity base, in the same way as has been done for cable stability, also offer the potential for cost reductions.
floating systems	floating turbines can reduce OpEx costs significantly by widening access windows and removing reliance on expensive dynamic positioning vessels to carry out offshore operations. Devices can instead be recovered to port or calmer waters for maintenance activities. Floating devices also allow deeper water sites to be developed. OpEx is estimated to account for 17% of total project costs for floating devices, compared with 43% for seabed-mounted devices [118]. 2012 OpEx estimates by Orbital Marine Energy for a prospective floating array using five 2 MW devices range between 0.16 and 0.24 m£/MW/year (excluding decommissioning), with an expectation for OpEx to rapidly reduce to below 0.1 m£/MW/year on subsequent projects [123]. For comparison, the OpEx of the MeyGen 1A seabed mounted turbines is 0.23 m£/MW/year.
multi-rotor systems	multi-rotor systems provide constructive interference between rotors, with experimental data showing a 20% power performance increase with a 10% thrust increase owing to this local blockage [124]. Initial LCoE estimates show that this can result in a *ca* 10% cost of energy reduction.
array optimization	gradient-based algorithms have been implemented within 2D hydrodynamic models to optimize the number of turbines and their position within an array [40–42]. Results demonstrate that mean turbine power can increase by up to 30% relative to a regular array layout, thereby significantly reducing LCoE.

### (d) Learning

The experience gained from all aspects of a project can lead to significant cost savings through better understanding of processes and design [4]. The learning from MeyGen phase 1A has been captured in three reports covering the design, installation and operating phases of the project [19,125,126]. While there are too many lessons to list all of them here, we summarize some of the most significant ones in table 5. These examples of learning, along with the other cost reduction drivers demonstrated in this appendix, all contribute to the ‘technology learning rate’, which is discussed and implemented in §3 to provide LCoE projections.

**Table 5.** Summary of learning from MeyGen phase 1A.

mechanism	description
turbine design	MeyGen phase 1A used two different turbine suppliers. While it has been acknowledged that this increased overall cost relative to using a single turbine supplier, it allowed the two suppliers to learn from one another through comparison of different techniques and equipment to steepen the learning curve.
	an improved understanding of site conditions such as turbulence was gained through multiple acoustic Doppler current profiler measurement campaigns, leading to an improved understanding of turbine loading to inform future turbine design. This is complemented by recent research that characterizes turbine loading [127–129].
offshore operations	during offshore operations MeyGen gained an improved understanding of the ability of different vessels to undertake specific tasks, informing future decision making on vessel selection to de-risk operations. It was also found that, in some cases, two marine operations could be performed in a single neap tide period, reducing vessel time and cost.
turbine micro-siting	it proved challenging to find suitably flat regions of the site to micro-site the turbines using gravity-based foundations and support structure, highlighting the value of monopile structures for future development, which de-risk offshore operations and enable turbines to be positioned in higher energy regions of a site.
power performance	learning from power performance analysis of the MeyGen array shows that the turbines are operating with a power coefficient of 0.41, 8% higher than their target power coefficient of 0.38. This learning can be used to inform projected energy yield estimates for future phased development.

### (e) Financing

The CapEx of tidal stream energy projects is relatively high, typically requiring finance from a combination of grant funding, debt and equity. Attracting finance requires high levels of investor confidence. Given the relative infancy of the industry, and therefore high risk, this can be hard to achieve. Projects using more established technologies such as onshore/fixed-bed offshore wind or solar PV offer lower risk investments, since years of operational experience allow risks to be more accurately quantified and mitigated against. In recent years, tidal stream energy projects have become operational. Detailed array performance is now being reported publicly on projects such as MeyGen 1A [19]. To date, the tidal stream energy industry has accumulated approximately 1.4 million operating hours. During this time, there has been a reduction in the empirical failure rate and the likelihood of failure similar to that experienced by the wind industry at a similar stage [10]. Through the ongoing demonstration of operational arrays, and investor confidence, the cost of capital is expected to reduce. However, of the operational projects to date, only the MeyGen project has publicized power performance and cost data. So while operational data are being gathered, there is poor visibility over how operational assets are performing. The ongoing demonstration and performance reporting of operational projects is expected to reduce cost of capital as investor confidence in the technology increases.

The increase in operational hours can also increase the range of insurance products for tidal stream energy. Through increased operational experience gained by site operators, insurance claims become better understood, which can lead to a reduction in premiums. Technology innovations such as sub-sea hubs and wet-mate connectors reduce the need for cable lifting operations, reducing risk of cable failure. Cable failures in fixed-bed offshore wind accounted for 77% of the total global cost of fixed-bed offshore wind farm losses in 2015 [118].
